# Supplementary material for: Development of New Edible Biodegradable Films Containing Camu-Camu and Agro-Industry Residue
Source: Polymers (Basel). 2024 Jun 27;16(13):1826. doi: 10.3390/polym16131826 (PMC11243893; doi:10.3390/polym16131826)
Supplement: Supplementary file 1 [file polymers-16-01826-s001.zip › polymers-2986574-supplementary.pdf]

polymers

MDPI

Supplementary Materials - Photographs of Sample 04 and Gelatine Agmag Sample 04 after 15 and 30 days of degradation, respectively.

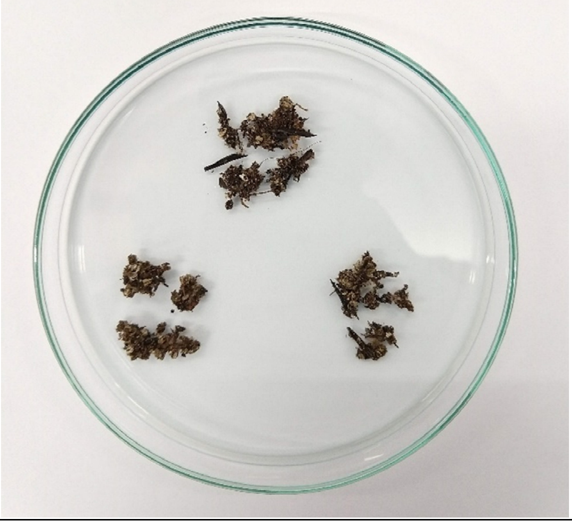

Sample 04 – 15 days

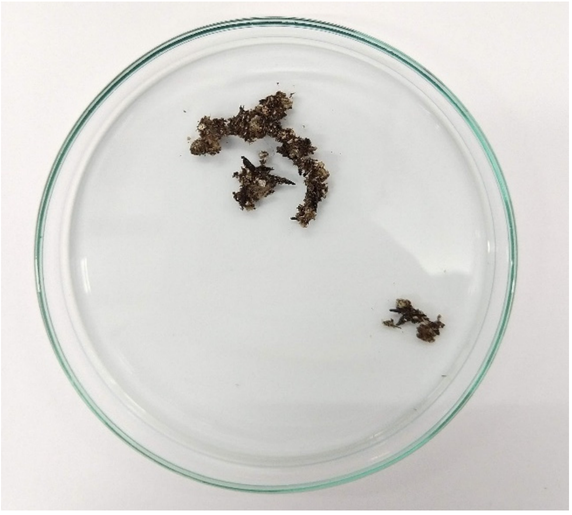

Gelatine Agmag Sample 04 – 15 days

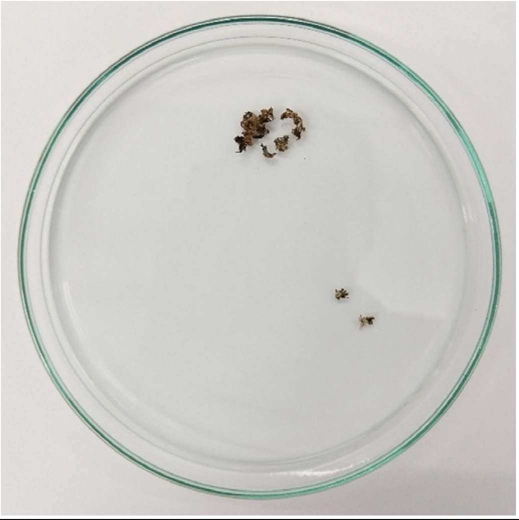

Sample 04 – 15 days

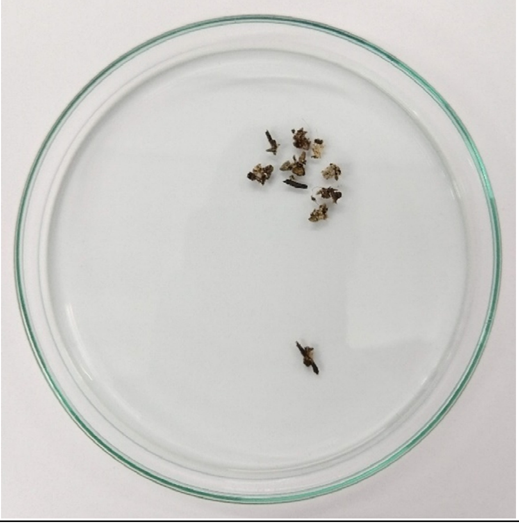

Gelatine Agmag Sample 04 – 15 days
